# Accuracy of the Measurement of Uterine Leiomyoma by Transabdominal Ultrasonography

**DOI:** 10.7759/cureus.68193

**Published:** 2024-08-30

**Authors:** Kenta Oue, Makiko Matsuda, Tomoyuki Ichimura, Makoto Murakami, Naoki Kawamura, Toshiyuki Sumi

**Affiliations:** 1 Department of Obstetrics and Gynecology, Osaka City University Graduate School of Medicine, Osaka, JPN; 2 Department of Gynecology, Izumi City General Hospital, Osaka, JPN; 3 Department of Obstetrics and Gynecology, Osaka City General Hospital, Osaka, JPN; 4 Department of Obstetrics and GynecologyGynecology, Osaka City General Hospital, Osaka, JPN; 5 Department of Gynecology, Nishi-Umeda City Clinic, Osaka, JPN; 6 Department of Obstetrics and Gynecology, Osaka Metropolitan University Graduate School of Medicine, Osaka, JPN

**Keywords:** intraclass correlation coefficient, conservative management, mri, transabdominal ultrasonography, uterine leiomyoma

## Abstract

Introduction

Uterine leiomyoma is a benign smooth muscle tumor. It does not necessarily require curative treatment, but if conservative management is chosen, it is important to rule out uterine leiomyosarcoma. When a size increase is observed, one must consider malignancy, and thus objective and cost-effective measurement of uterine size is important, especially for early detection of malignant change. Although MRI imaging is thought to be the gold standard for the diagnosis of uterine leiomyosarcoma, frequent MRI is impractical because of the incidence of uterine leiomyoma and the economic burden in real-world clinical practice. On the other hand, ultrasonography (US) is considered the most useful device in the observation of size changes. So this study aimed to examine the accuracy of the measurement of transabdominal US compared to MRI imaging.

Materials and methods

This retrospective study included 92 patients with uterine myoma ≥ 50 mm who undertook an MRI within 30 days after the transabdominal US. The longest diameter of the largest myoma (a), the longest diameter perpendicular to a in the sagittal image (b), and the longest diameter perpendicular to a and b in the axial image (c) were measured by US and MRI, and these were used to calculate the volume. Results were analyzed by intraclass correlation coefficient (ICC) 3.1.

Results

The ICC for the volume and major axis of the largest myoma by US and MRI were 0.87 and 0.90, respectively. The 95% confidence intervals (CI) were 0.82-0.91 and 0.87-0.93, respectively. Both reliability levels ranged from good to excellent. ICC was 0.54 (95%CI 0.15-0.78) in myomas with a volume of >500 cm^3^, and the concordant rate between US and MRI was poor to good. On the other hand, ICC was 0.82 (95%CI 0.57-0.93) even though all myomas with major axes greater than 120 mm had a volume >500 cm^3^, and the concordant rate between US and MRI measurements was moderate to excellent. In the evaluation by major axis, ICC was 0.60 (95%CI -0.41-0.95) for myomas larger than 160 mm, indicating a lower concordant rate.

Conclusion

Transabdominal US is an appropriate modality as well as MRI for follow-up of uterine myoma size if the nodules are 160 mm or smaller. Measurement of the major axis is easier and more useful than volume.

## Introduction

Uterine smooth muscle tumors are common pelvic tumors, and the most common are uterine leiomyoma (UL), a benign tumor. Since ULs generally shrink spontaneously after menopause, not all myomas require curative treatment. There are various strategies for the treatment and management of ULs, including curative treatment such as hysterectomy and myomectomy, surgical treatment such as uterine arterial embolization, microwave endometrial ablation, and focused ultrasound surgery, hormone therapy, and symptomatic treatment with follow-up [1,2]. Except for hysterectomy and myomectomy, no histopathological examination of myoma will be performed in either direction.

In cases where a conservative policy without tumor removal is chosen, it is important to rule out uterine leiomyosarcoma. Uterine leiomyosarcoma is a rare disease that reportedly accounts for about 1% of all uterine malignancies, but its prognosis is poor [3,4]. There have also been reports of malignant transformation of tumors thought to be ULs into uterine leiomyosarcoma [5], and there are also reports that mutations in UL driver gene *MED12* are also found in uterine leiomyosarcomas [6]. The definitive diagnosis of UL and uterine leiomyosarcoma is made by histopathologic diagnosis, but the most useful clinical differentiation is based on the appearance of high-signal areas and postmenopausal enlargement or rapid enlargement on magnetic resonance imaging (MRI) [7].

Repeated MRI can provide information on changes in the internal nature and size of the tumor. However, the incidence of uterine leiomyosarcoma and medical economics considerations make frequent MRI impractical. On the other hand, ultrasonography (US) takes lower cost and less time. US is considered the most useful device in the observation of size changes. To assess the rapid growth or postmenopausal growth of tumors, which is one feature of leiomyosarcoma [8], accurate measurement is important. There are a few studies that reported the measurement of uterine myoma by US vs MRI. In past studies, transvaginal US was used in most cases [9-11]. To our knowledge, there are no previous studies about the measurement of myoma with transabdominal US compared to MRI. 

In this study, we examined whether transabdominal US is an appropriate modality to observe size changes of UL by comparing the measurement of transabdominal US with MRI, and whether transabdominal US could be a substitute for measurement of uterine myoma size by MRI.

This article was previously presented as a meeting abstract at the 66th Annual Congress of the Japan Society of Obstetrics and Gynecology on April 18-20, 2014.

## Materials and methods

Sample selection

The study included 92 consecutive outpatients attending the outpatient clinic specializing in uterine myoma at Osaka City University Hospital, Osaka, Japan, from July 2005 to July 2013, who had myoma with a long diameter of ≥ 50 mm and undertook MRI within 30 days after they were performed transabdominal US. Patients who had myoma with unclear margins on MRI, and who were undergoing or just completed hormone therapy were excluded. The study was approved by the Ethics Committee of Osaka City University (approval number: 2959).

Methods

**Figure 1:**
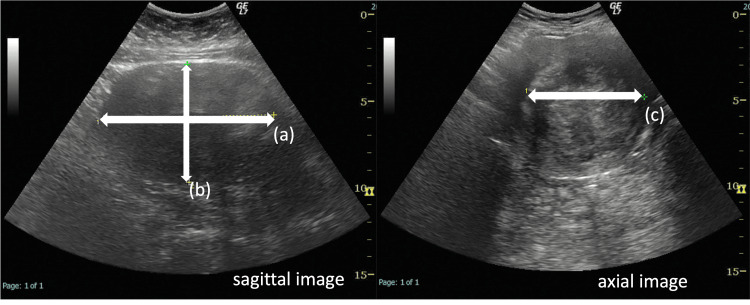
Schema of measurement of three axes of a myoma nodule by ultrasonography a: longest diameter of myoma in the sagittal image; b: longest diameter perpendicular to “a”; c: longest diameter perpendicular to “a” and “b” in the axial image

A retrospective study was performed to determine the measurement error regarding the volume and longest diameter of the largest size nodule as measured by MRI and US. The major diameter and volume of the largest myoma in each case with transabdominal US and MRI were measured. Measurement using US was performed by a single experienced gynecologist with LOGIQ7 (GE HealthCare Technologies, Inc., Chicago, Illinois, United States). He measured and recorded the longest diameter of myoma in sagittal image (a), the longest diameter perpendicular to “a” (b), and the longest diameter perpendicular to “a” and “b” in axial image (c) (Figure 1).

MRI images were evaluated by a single radiologist with expertise in MRI for gynecologic tumors. MRI was performed with 1.5 Tesla scanners (Avanto; Siemens AG, Munich, Germany). Sagittal and axial T2-weighted MRI scans without contrasts were evaluated for the location and size of the myoma. The diameters of the largest-sized myoma were measured and recorded as in the US studies. The volume of ULs was calculated by applying the ellipsoid formula (volume=4/3π×a/2×b/2×c/2). To determine whether menstruation affects the occurrence of measurement error, we also examined whether there is a significant difference between MRI and US measurements in postmenopausal and premenopausal patients. The absolute value of error in measuring the length diameter of the same nodule by MRI and US was calculated, and the error in the postmenopausal group was defined as PE and the error in the premenopausal group as BE.

Statistical analysis

The results of measurement by transabdominal US and MRI were analyzed by Intraclass correlation coefficient (ICC) 3.1 with R version 4.2.3 (R Foundation for Statistical Computing, Vienna, Austria). In this study, ICC interpretation was evaluated based on 95% confidence intervals (CIs) of ICC estimates using the following general guidelines. Values less than 0.50: poor reliability, values between 0.50 and less than 0.75: moderate reliability, values between 0.75 and less than 0.90: good reliability, and values 0.90 or more: excellent reliability. When the 95% CI straddled the moderate reliability range and the good reliability range, the confidence level was determined to be moderate to good [12]. Significant difference tests for measurement error in postmenopausal and premenopausal cases were performed using Mann-Whitney's U test.

## Results

The clinical features of the patients are summarized in Table 1. The patients’ age ranged from 28 to 72 years with a median age of 48 years. A total of 19 patients were postmenopausal. MRI measurements of the volume of the largest nodule ranged from 46 to 1438 cm^3^ with a median volume of 263.5 cm^3^, while those by US ranged from 49 to 1498 cm^3^ with a median volume of 254 cm^3^. MRI measurements of the major diameter of the largest nodule ranged from 50 to 218 mm with a median length of 91 mm, and those by US ranged from 47 to 203 mm with a median length of 88 mm. The major diameter in two-thirds of the tumors was under 100 mm. The volume of the largest nodule in 49 cases was ≥ 250 cm^3^. About one-fourth of the cases had single nodules. The location of the largest nodule was not specific.

**Table 1:** Characteristics of 92 patients *including 2 cases less than 50 mm Number of postmenopause cases=19 (21%) US: ultrasonography

Charcteristics		
Age median (years old)	48 (28-72)	
	Measurement by MRI	Measurement by US
Major axis of the largest myoma (mm)		
50-99 mm	62 cases (67%)	59 cases (64%) *
100-149 mm	25 cases (27%)	29 cases (32%)
≧150 mm	5 cases (5%)	4 cases (4%)
Volume of the largest myoma (cm^3^)		
<250	43 cases (47%)	45 cases (49%)
250-499	28 cases(30%)	27 cases (29%)
500-749	11 cases(12%)	9 cases (10%)
750-999	5 cases (5%)	8 cases (9%)
≧1000	5 cases (5%)	3 cases (3%)

**Figure 2:**
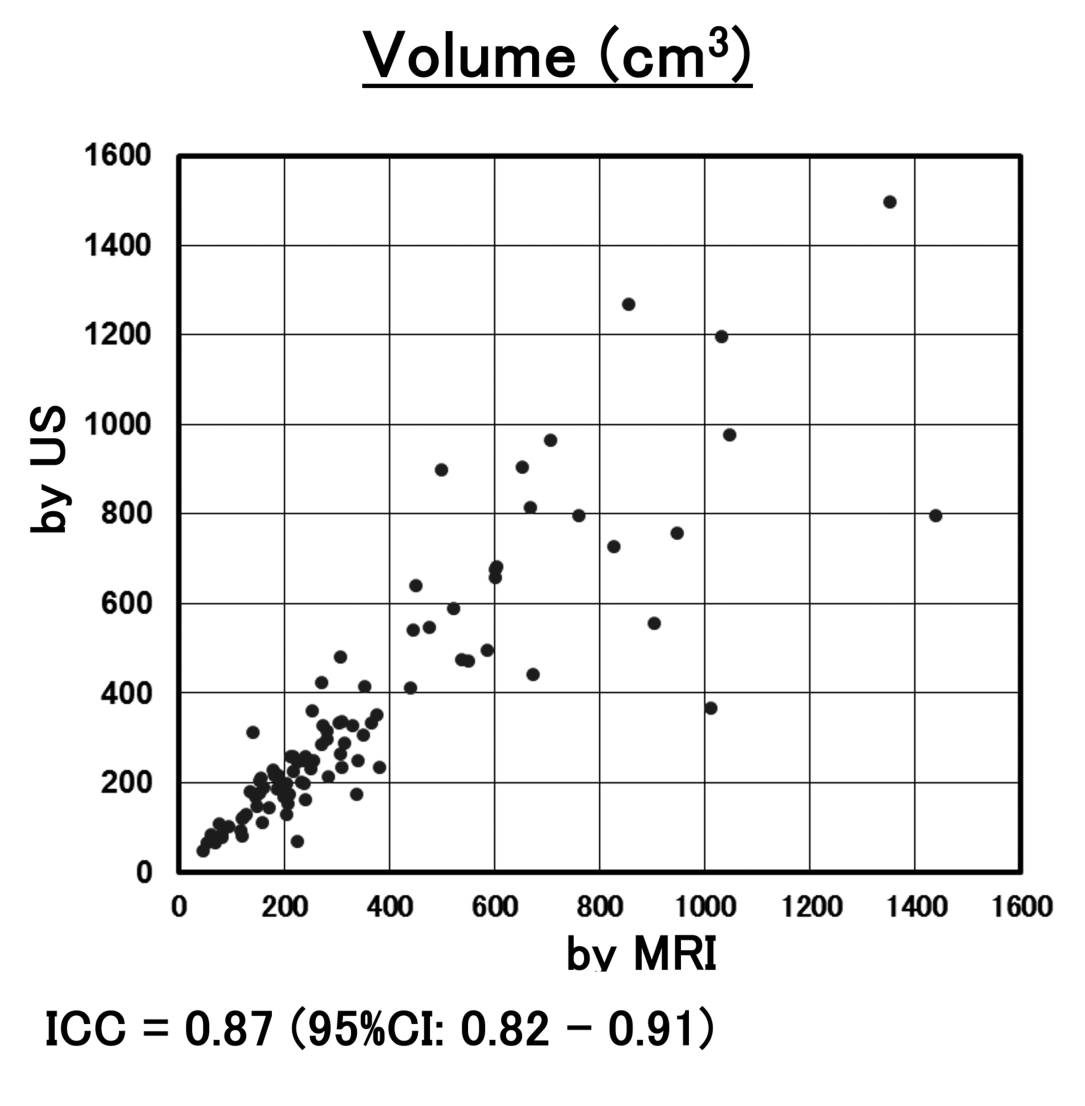
The results of myoma volume measurements The results were plotted for each case based on volume measured by US (y-axis) and volume measured by MRI (x-axis). ICC for the volume of myoma by US and MRI was 0.87 (95%CI: 0.82 - 0.91). US: ultrasonography; ICC: intraclass correlation coefficient

**Figure 3:**
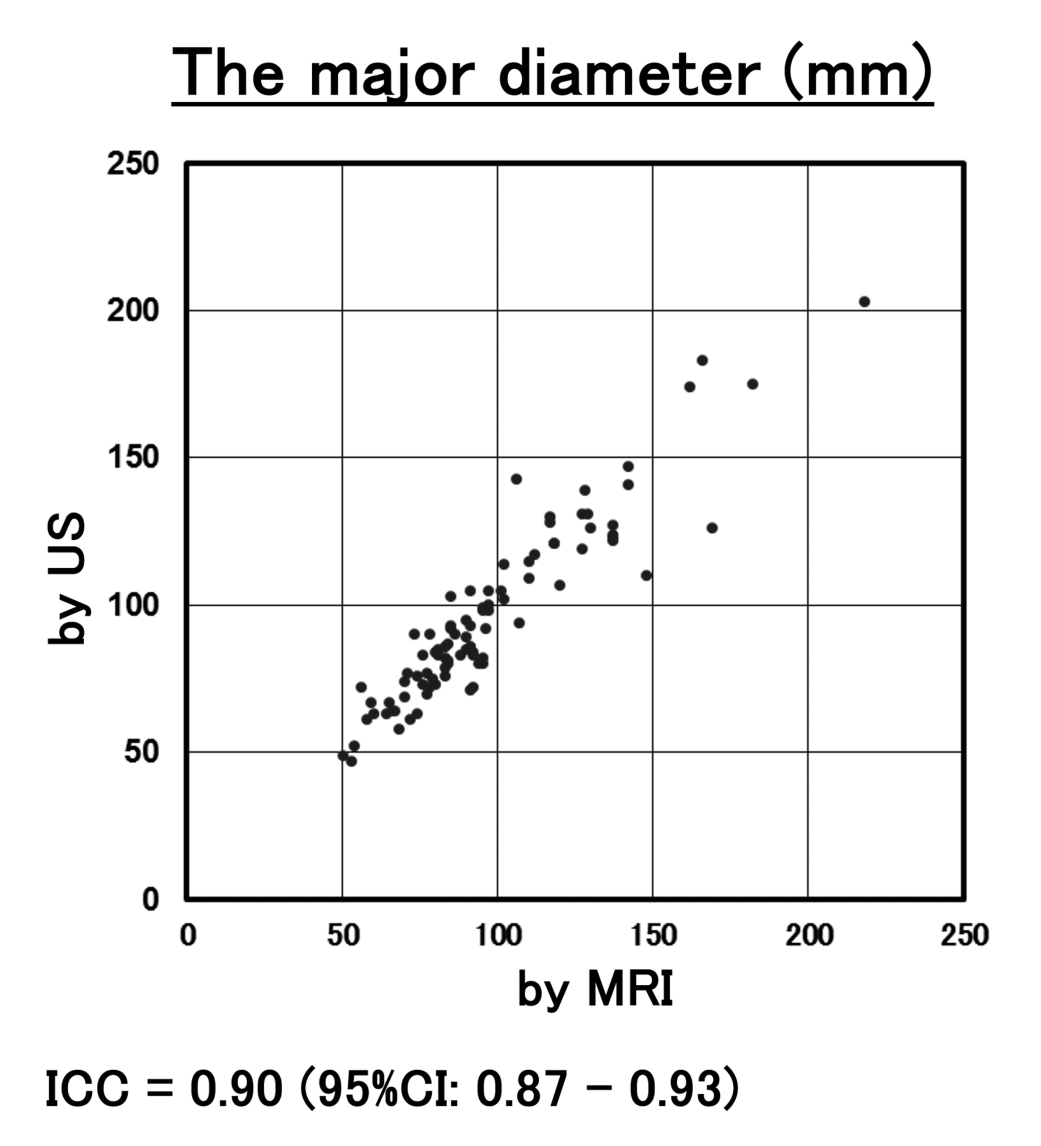
The results of myoma major axis measurements The results were plotted for each case based on length measured by US (y-axis) and length measured by MRI (x-axis). ICC for the major diameter of myoma by US and MRI was 0.90 (95%CI: 0.87 - 0.93). US: ultrasonography; ICC: intraclass correlation coefficient

**Figure 4:**
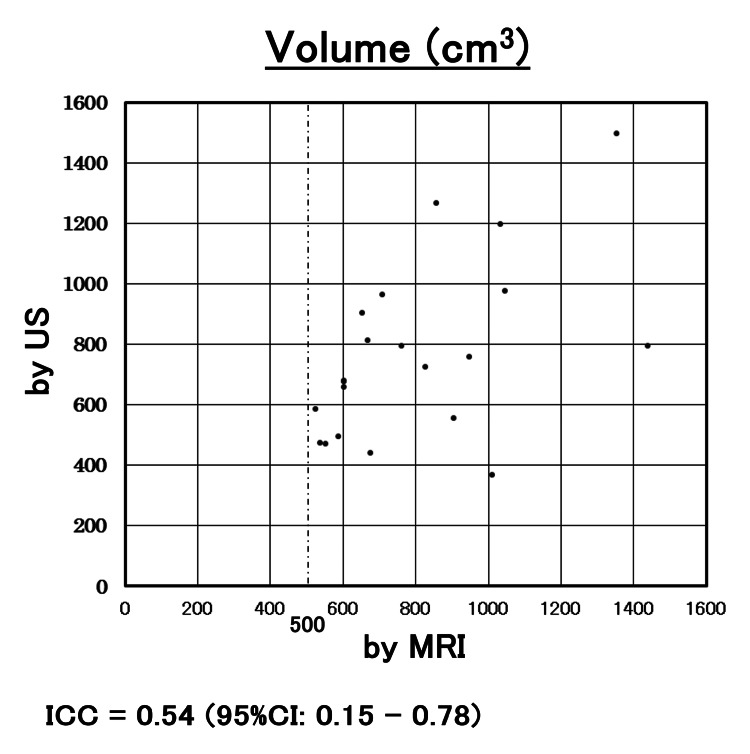
Myoma volume measurements for nodules with a volume greater than 500 cc This shows the results of myoma volume measurements for nodules with a volume greater than 500 cm^3^, which were plotted for each case based on volume measured by US (y-axis) and volume measured by MRI (x-axis). ICC for the volume of myoma by US and MRI was 0.54 (95%CI: 0.15 - 0.78). US: ultrasonography; ICC: intraclass correlation coefficient

**Figure 5:**
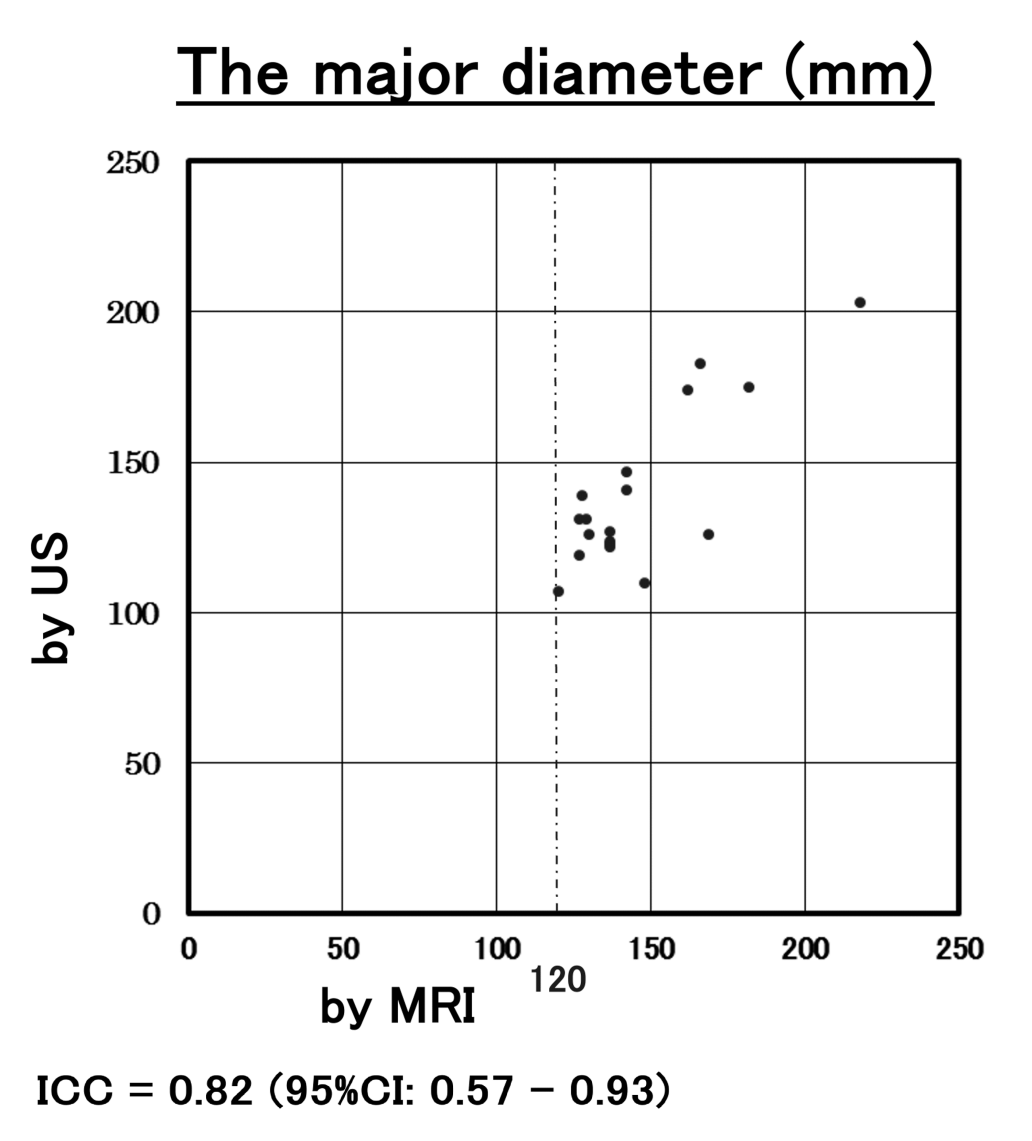
Myoma major axis measurements for nodules with a length greater than 120 mm This shows the results of myoma major axis measurements for nodules with a length greater than 120 mm, which were plotted for each case based on length measured by US (y-axis) and length measured by MRI (x-axis). ICC for the major diameter of myoma by US and MRI was 0.82 (95%CI: 0.57 - 0.93). US: ultrasonography; ICC: intraclass correlation coefficient

**Figure 6:**
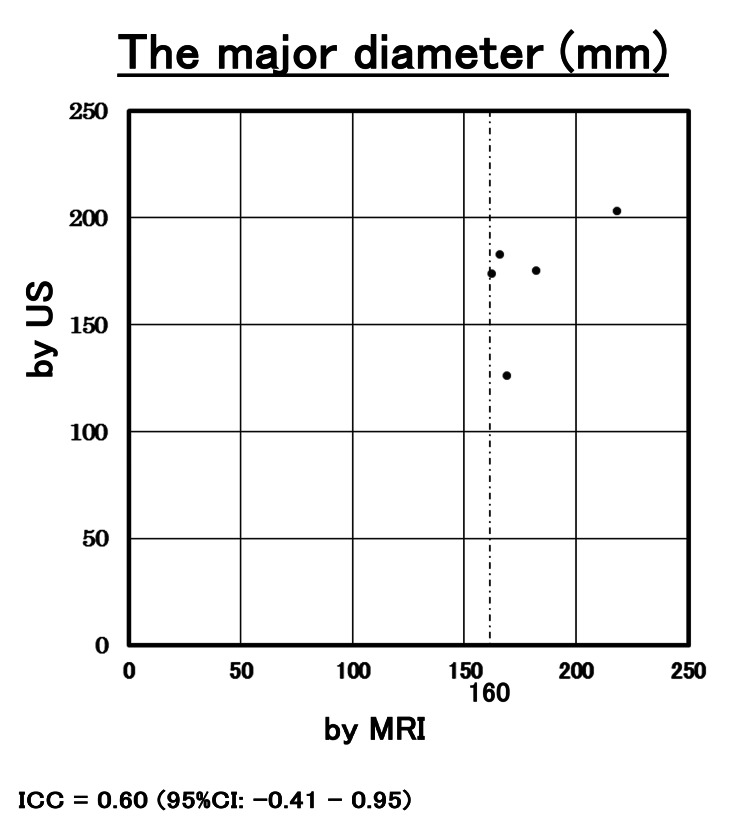
Myoma major axis measurements for nodules with a length greater than 160 mm This shows the results of myoma major axis measurements for nodules with a length greater than 160 mm, which were plotted for each case based on length measured by US (y-axis) and length measured by MRI (x-axis). ICC for the major diameter of myoma by US and MRI was 0.60 (95%CI: -0.41 - 0.95). US: ultrasonography; ICC: intraclass correlation coefficient

**Figure 7:**
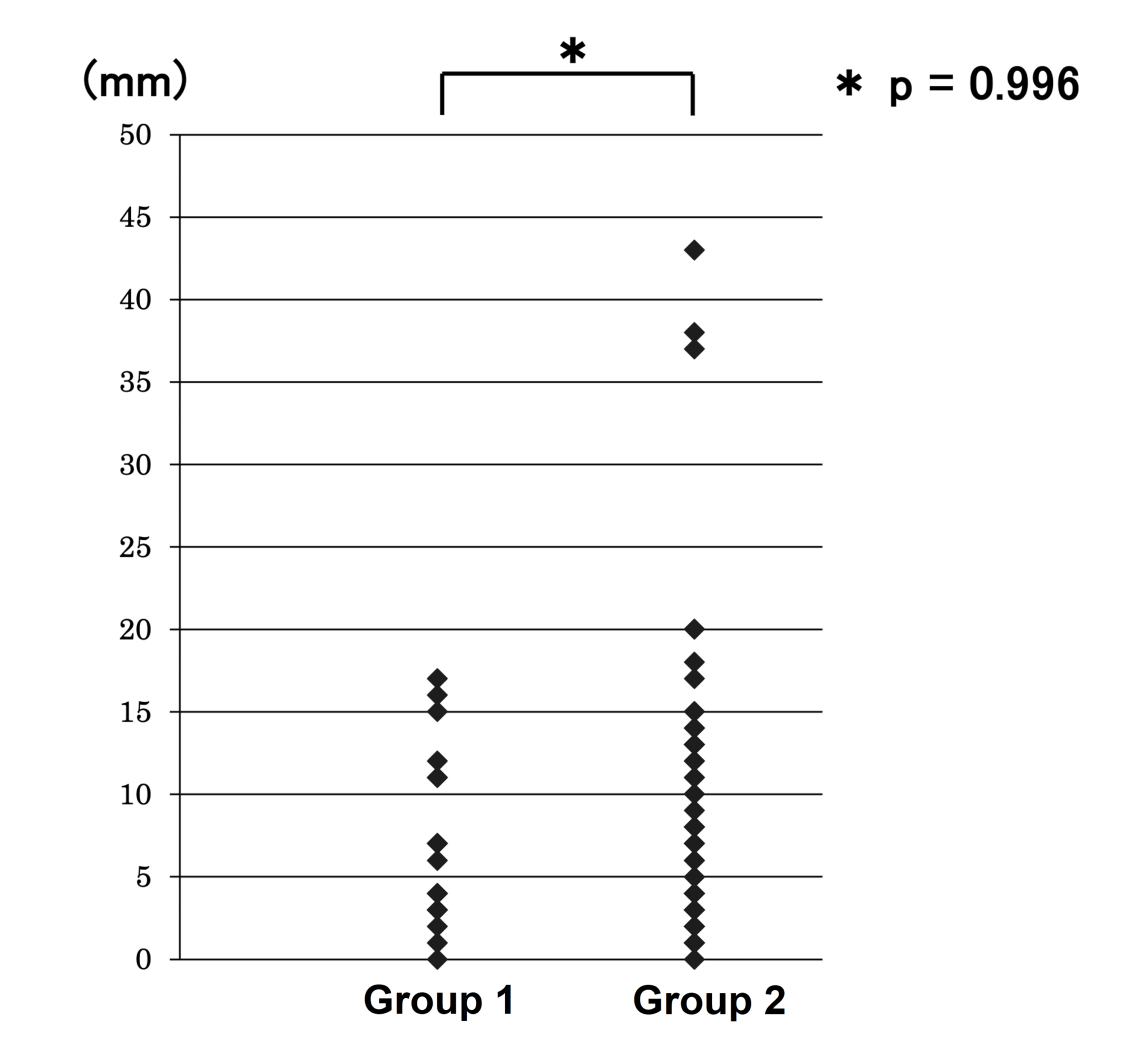
The absolute values (vertical axis) of the difference in myoma major axis measurements by MRI and US This graph shows the absolute values (y-axis) of the difference in myoma major axis length (mm) measurements by MRI and US in the postmenopausal group (Group 1) and the premenopausal group (Group 2), with no significant difference in the median values between the two groups (p = 0.996). US: ultrasonography; Group 1: postmenopausal group; Group 2: premenopausal group

The correlation of the measurement by US with MRI in volume and the major diameter is shown in Figures 2, 3. ICC for the volume and major diameter of the largest myoma by US and MRI were 0.87 and 0.90, respectively. The 95% CIs were 0.82-0.91 and 0.87-0.93, respectively. Both reliability levels ranged from good to excellent. In this study, ICC was 0.54 (95%CI 0.15-0.78) in myomas with a volume of > 500cm^3^, and the concordant rate between US and MRI was poor to good (Figure 4). On the other hand, ICC was 0.82 (95%CI 0.57-0.93) in myomas with major diameters greater than 120 mm, and the concordant rate between US and MRI measurements was moderate to excellent (Figure 5). In the evaluation by major diameter, ICC was 0.60 (95%CI -0.41-0.95) for myomas larger than 160 mm, indicating a lower concordant rate (Figure 6). The median PE was 6 mm (0-17 mm) and the median BE was 5 mm (0-43 mm). There was no significant difference between median PE and BE (p=0.996) (Figure 7).

## Discussion

In the conservative management of ULs, it is important to differentiate them from uterine leiomyosarcomas, which resemble ULs. One of the clinical differences between ULs and leiomyosarcomas is the augmentation speed [8]. Accurate size measurement is important to determine the rate of enlargement, and MRI scans can provide an accurate measurement of myoma size. However, it is not practical to perform frequent MRIs during follow-ups of ULs, and US is usually used for measurements. If myoma size is measured by US rather than MRI, it would be important to understand the degree of error in the measurement results from the two types of devices. A few studies reported a comparison of the measurement of uterine myoma by US vs MRI [9-11], but almost all of them used transvaginal US. Dueholm et al. reported that MRI and transvaginal US were equally highly accurate in ascertaining the presence of myoma but that MRI was superior to transvaginal US for the mapping of individual myoma, and although the efficacy of MRI did not depend on the uterine volume, transvaginal US efficacy became significantly poorer than that of MRI at volumes >375 mL [10]. In this study, we investigated the reliability and validity of transabdominal US measurements of UL volume and major diameter using MRI measurements as the gold standard. Small ULs less than 50 mm in length do not need to be differentiated from uterine sarcomas [13], and most of them are followed up in the clinic. Few myoma cases with a major diameter of less than 50 mm have been followed up in our hospital and, therefore, the subjects included in this study were patients with myoma nodules with a major axis of 50 mm or more as measured by MRI. In addition, the measurement of UL by US was performed transabdominally to accommodate large nodules.

The results of this study showed the validity of US measurement of both myoma volume and uterine length diameter. However, in cases where the volume exceeded 500 cm^3^, the ICC was 0.54, which did not indicate the validity of the US measurements. More than 90% of cases with a volume of more than 500 cm^3^ in this study had a major diameter exceeding 120 mm but ICC for cases with major diameter of more than 120 mm was 0.82, suggesting the validity of major diameter measurement by US. But even in the measurement of major diameter, ICC was 0.60 for diameters greater than 160 mm, and the concordance rate between US and MRI was lower. These results indicate that the concordance rate with MRI is high for the measurement of UL by US in measuring major diameters for nodules of 160 mm or less. 

It is not known how the size increase occurs when the myoma becomes malignant, whether it increases uniformly or only in a certain direction, or whether it changes in the same way in all cases. Measuring the longest diameter does not necessarily recognize myoma malignant transformation. However, we believe that this measurement is likely to be useful in the diagnosis of leiomyosarcoma because it is likely that at least a certain amount of the myoma's longest diameter will increase. We expected the premenopausal patients to have a larger measurement error, because of the influence of menstrual cycles. However, in this study, there was no significant difference between PE and BE. It should be noted that measurement errors exceeding 20 mm were observed only in the premenopausal group, and it cannot be said with certainty that there is no influence of measurement errors associated with menstruation. It is necessary to consider the timing of measurement in premenopausal patients by either measuring hormone levels or measurement adjusted for the menstrual cycle.

How can this study be applied to daily clinical practice? When following UL for a long term, patients are often sensitive to changes in size, and a sudden increase in size should also raise the possibility of leiomyosarcoma. MRI is considered most appropriate for accurate identification of myoma size and differentiation of leiomyosarcoma. However, frequent MRIs are not practical. Transabdominal US can be performed more frequently than MRI and may be able to detect size changes earlier. This study suggests that transabdominal US may be useful in the follow-up of myomas up to 160 mm in major diameter. However, MRI may be more appropriate for the definitive diagnosis when following myomas with a major diameter greater than 160 mm or when transabdominal US results are suspicious for leiomyosarcoma.

There are some limitations of this study. The US myoma measurements were performed by the same gynecologist. We believe that the validity of transabdominal ultrasound for major axis length measurement was demonstrated under these conditions. On the other hand, when measuring the size of uterine myoma by US, it is expected that the boundary of the nodule will be different depending on the examiner, and if the examiners are not the same, the results will unfortunately not be the same as in the present study. Additionally, the difficulty of measurement of large myoma was caused by the mechanical limitation of US. Large myomas must be measured by dividing into two pieces, so the measurement often becomes inaccurate. This fact leads to inaccuracy of measurement in myoma sized over 160 mm. MRI is likely needed for accurate measurement of myomas larger than 160 mm.

However, to the best of our knowledge, this study was the first to examine uterine myoma size measured by transabdominal US compared to MRI.

## Conclusions

Transabdominal US is an appropriate modality as well as MRI for follow-up of uterine myoma size if the nodules are 160 mm or smaller. Measurement of major diameter is easier and more useful than volume. It is still unclear how useful follow-up using only the major diameter, rather than volume, is for diagnosing leiomyosarcoma. However, US can be performed more frequently than MRI and is a useful tool for monitoring changes in size. This study could be useful for patients and gynecologists who have difficulty accessing MRI for various reasons.
